# Measuring Urinary Sensation with Current Perception Threshold: A Comparison between Method of Limits and Method of Levels

**DOI:** 10.1155/2012/868915

**Published:** 2011-10-20

**Authors:** Carley Davis, Lior Lowenstein, Elizabeth Mueller, Linda Brubaker, Kimberly Kenton

**Affiliations:** ^1^Division of Female Pelvic Medicine and Reconstructive Surgery, Department of Urology, Loyola University Medical Center, USA; ^2^Department of Obstetrics and Gynecology, Rambam Health Care Campus, 8 Ha'Aliya Street, Haifa 31096, Israel

## Abstract

*Purpose*. To determine the association between the two methods of obtaining current perception thresholds (CPTs) in the lower urinary tract (LUT). *Materials and Methods*. Twenty-one women undergoing pelvic surgery underwent CPT determinations of the urethra. CPTs were measured at 2,000, 250, and 5 Hz (corresponding to A-*β*, A-*δ*, and C fibers, resp.) both pre- and postoperatively. Threshold values were obtained in all patients by using the method of limits and the method of levels. *Results*. CPT values obtained by using the method of levels and the methods of limits were highly correlated at all frequencies before and after surgery (*ρ* = 0.93–0.99, *P* < 0.0001). The mean threshold values obtained by the method of levels were significantly lower at all frequencies compared with those obtained by the method of limits. *Conclusions*. Our findings suggest that the method of levels is more sensitive for the detection of CPTs compared to the method of limits.

## 1. Introduction

Given the high-quality evidence supporting the role of afferent innervation in LUT dysfunction, it is essential to validate clinical methods that quantify afferent nerve function. The two most common methods which are currently used to assess CPT's are the method of levels and the method of limits. When reviewing the literature, we found that there have been several studies reporting the normative CPT data in the lower urinary tract [1–6]. Depending on the institution, different techniques and methods are being used to collect this normative data. Based on this established normative data, studies are now focusing on using CPTs in pathologic states [7, 8]. Unless the collection of data is standardized, it will become increasingly difficult to compare or reproduce studies. 

Afferent innervation of the lower urinary tract and the vaginal area can be assessed with electrodiagnostic testing. CPT measurement using the Neurometer is a standard technique used to assess the function of afferent sensory nerves [9, 10]. The Neurometer is a constant current stimulator which selectively measures and quantifies different size of sensory nerve populations. Afferent neurons are depolarized by different frequency sine waves depending on their membrane ion channel concentration. This allows differentiation between the major types of afferent neurons based on the frequency of neural stimulation. Large myelinated A-*β* fibers are stimulated at 2000 Hz, smaller myelinated A-*δ* fibers are stimulated at 250 Hz, and unmyelinated C fibers are stimulated at 5 Hz. 

The Neurometer can be used to obtain CPT's by using either the method of limits or the method of levels. The method of limits uses the manual function of the Neurometer to increase the stimulus until the patient can perceive it for the first time, the upper limit. It is then decreased until the stimulus is no longer perceived, the lower limit. The upper and lower values are averaged to obtain the CPT value. The method of levels uses the automated function of the Neurometer where the patient is put through a series of forced choice tests. True and false stimuli are given in an arbitrary order, and the patient indicates which stimulus is true. If answered correctly, the next presented stimulus is of a lower intensity level. When using the method of levels, the determination of the threshold is based on the lowest stimulus level which the patient correctly detects 50% of the time. 

In the current study, we evaluated the association between the two most commonly used methods for obtaining CPT values in the lower urinary tract.

## 2. Materials and Methods

After approval by our Institutional Review Board, we consecutively enrolled patients from our clinic who were planning on having pelvic reconstructive surgery between September 2006 and May 2007. All women underwent a standardized clinical evaluation including history, physical, and gynecological examination. Our exclusion criteria included: patients with any neurologic disorder or neuropathy, a postvoid residual volume greater than 150 mL with no evidence of pelvic organ prolapse and patients with cognitive impairment. After signing an informed consent, participants underwent CPT testing preoperatively. On postoperative day one or two, the CPT testing was repeated at the patient's bedside.

### 2.1. CPT Protocol

A ring electrode was positioned 1 cm distal to the balloon of a 14 Fr foley catheter which was placed in the subject's urethra. The balloon was inflated and the catheter was pulled snug to assure the electrode was in the urethra. Any residual urine was drained and continued to drain throughout the testing. 

Subjects underwent CPT testing in a standardized fashion using a Neurometer CPT device in the dorsal lithotomy position. The 2000 Hz frequency was tested first using the method of limits technique. The amplitude was slowly increased until the stimulus was perceived. This was recorded as the upper limit. The stimulus was turned off until the initial sensation subsided. The same stimulus was then slowly decreased until the patient no longer perceived the stimulus. The last stimulus the patient could perceive was termed the lower limit. The upper and lower limits were averaged to obtain the sensory threshold by the method of limits. The subject was then given a series of forced choice tests by the Neurometer to determine the sensory threshold by the method of levels starting at the lower limit obtained by the method of limits. The Neurometer randomly picks real and false stimuli separated by a 3–5 second rest period. The subject indicated which stimulus was stronger as the intensity was decreased by 0.4 *μ*A increments. Both the method of limits and the method of levels were then repeated at 250 Hz and 5 Hz.

### 2.2. Statistical Analysis

SPSS for Windows version 16 (Chicago, IL, USA) was used for data management and statistical analysis. CPT values were reported in mA using both the mean and standard deviation. The Wilcoxon Signed Rank was used to compare noncategorical parameters. The correlation between the thresholds obtained by the methods of limits and the method of levels was assessed by Spearman's correlation test. All tests were considered significant at the 0.05 level. No one-sided tests were done.

## 3. Results

Twenty-one women with a mean age of 59 ± 12 years participated in the study. The majority of the patients were Caucasians 90% (19) and the rest were Hispanic. Demographic and medical history information is listed in Table 1. 

CPT values obtained by the method of levels were significantly lower at all tested frequencies compared with the values obtained by the method of limits (Table 2). These differences persisted both before and after surgery. Spearman's correlation demonstrated a significantly high correlation between the two methods of threshold evaluation, both before and after surgery at all frequencies (Spearman's rho ranges from 0.92 to 0.99, *P* < 0.001, Table 2).

## 4. Discussion

Our study is the first to evaluate the correlations between the two most common methods of CPT evaluation. Our results demonstrate that the threshold values obtained by the method of levels were persistently lower compared with the values obtained by the method of limits. There was a high correlation between the values obtained by the two different methods at all frequencies. These findings are supported by previous studies that compared the values of thermal threshold levels obtained by the method of levels to the threshold values obtained by the methods of limits. Similar to our findings, the threshold levels obtained by the methods of levels were consistently lower in both normal participants and patients with neuropathic compared with the values obtained by the method of limits [4, 7, 11, 12]. 

The role of CPT is becoming increasingly important in diagnosing abnormalities of afferent neural pathways which may contribute to pelvic floor disorders. Based on accumulating evidence, it seems likely that in certain pathological states in the pelvis and lower urinary tract, alternate afferent pathways are activated [13–15]. Currently the most common methods used in clinical practice and in published literature are CPT testing and QST using thermal and vibratory stimulation. CPT testing is the most commonly used method to quantify the functional integrity of specific afferent nerve fibers from the periphery to the central nervous system. Normative data for CPT in the LUT has been published in previous studies [1–6]. A review of the literature demonstrates significant variability in the testing equipment as well as inconsistencies in the methods used to obtain the LUT thresholds. The Neurometer device is commonly used in previously published studies [1–6]. This device offers two different, feasible and objective methods to measure LUT sensation. Manufacturer recommendations are that CPT testing with the Neurometer be done using the method of levels rather than the method of limits.

Though CPT threshold evaluation by the method of limits consumes less time, it seems to be less accurate compared with measurements obtained using the method of levels. A possible limitation to the use of the method of limits is the reaction time of the examinee. The reaction time is dependent on the conscious perception of the stimulus, processing of the information and generating an action to indicate a response. During this period of information processing before the subject indicates a response, the stimulus continues to increase or decrease leading to a deviation from the actual perceived stimuli. Another possible limitation to the method of limits technique is the nonstandardized rate of change of the intensity of the CPT stimulus. The examiner determines the rate at which the intensity both increases and decreases adding variability to the technique. The method of levels, being an automated series of forced choice tests, makes this method easy to reproduce and avoids possible inaccuracies due to subject reaction time and examiner variability.

## 5. Conclusion

In order to compare studies of LUT sensation, the method of data collection needs to be standardized. Our data demonstrates a high correlation between the method of limits and the method of levels using the Neurometer. The method of levels resulted in significantly lower CPT values. As a means of standardizing the data collection, we propose that the method of levels, with the above described technique, be instituted as the gold standard in measuring LUT sensory thresholds.

## Figures and Tables

**Table 1:** Patient demographics and medical history.

Age (years, median)	61% (31–79)
Race/Ethnicity (self-described)	
Caucasian	90% (19/21)
Hispanic	10% (2/21)
Hypertension	33% (7/21)
Estrogen treatment	20% (4/21)

Prior prolapse surgery	15% (3/21)
Prior hysterectomy	52% (11/21)
Prior incontinence surgery	23% (5/21)
Blood hypertension	36.8% (14/38)
Depression	18.4% (7/38)

Current surgery	
Sacrocolpopexy	38.1% (8/21)
Vaginal Hysterectomy + apical suspension	24% (5/21)
Colpocleisis	14% (3/21)
TVT	28% (6/12)
Suburethral fascial sling	14% (3/21)
Posterior repair	5% (1/21)

**Table 2:** Comparison and correlation between threshold levels obtained by methods of levels and methods of limits.

	Method of levels mean (STD)	Method of limits mean (STD)	^†^ *P*	Spearman's rho
Preoperative (mA)				
2000 Hz	1.70 (1.19)	2.09 (1.14)	0.0001	.934*
250 Hz	.65 (.37)	.80 (.40)	0.0001	.926*
5 Hz	.34 (.34)	.40 (.38)	0.008	.934*
Postoperative (mA)				
2000 Hz	2.70 (.17)	2.95 (1.72)	0.0001	.984*
250 Hz	1.41 (1.12)	1.60 (1.24)	0.0001	.988*
5 Hz	1.15 (1.60)	1.32 (1.62)	0.0001	.961*

^†^Wilcoxon Signed Rank Test; *P < 0.001.
